# Predictive Value of the Serum Cystatin C/Prealbumin Ratio in Combination With NT-proBNP Levels for Long-Term Prognosis in Chronic Heart Failure Patients: A Retrospective Cohort Study

**DOI:** 10.3389/fcvm.2021.684919

**Published:** 2021-07-14

**Authors:** Chuanhe Wang, Su Han, Fei Tong, Ying Li, Zhichao Li, Zhijun Sun

**Affiliations:** Department of Cardiology, Shengjing Hospital of China Medical University, Shenyang, China

**Keywords:** cystatin C, prealbumin, heart failure, long-term, mortality, prognosis

## Abstract

**Aim:** The present study was established to investigate the use of the serum cystatin C/prealbumin (Cys-C/PAB) ratio as a predictive factor for long-term prognosis in patients with chronic heart failure.

**Methods:** We divided our retrospective cohort of 6,311 patients admitted to hospital due to an episode of heart failure (HF) into three groups according to the Cys-C/PAB ratio. The endpoints were cardiovascular and all-cause mortality. Median follow-up time were 3.3 years (2–8 years), during which 2,945 (46.7%) patients died.

**Results:** The Cys-C/PAB ratio was revealed to be an independent predictor of cardiovascular mortality (HR: 1.12, 95% CI: 1.15–1.23, *P* < 0.01) and all-cause mortality (HR: 1.19, 95% CI: 1.13–1.24, *P* < 0.01) by multivariable Cox analysis. Integrated discrimination improvement (IDI) showed that the Cys-C/PAB ratio in conjunction with the level of N-terminal pro-B-type natriuretic peptide (NT-proBNP) conferred a significant improvement in predicting individual risks of cardiovascular (*P* = 0.023) and all-cause (*P* = 0.028) mortality. For those with a high Cys-C/PAB ratio in combination with a high NT-proBNP level, the long-term cardiovascular mortality risk ratio was 8.6-times higher than for those with low values, and 7.51-times for all-cause mortality. Our study also showed that Cys-C/PAB and NT-proBNP in combination displayed higher value for the prediction of cardiovascular and all-cause in-hospital mortality in patients with HF.

**Conclusions:** The Cys-C/PAB ratio is valuable for predicting cardiovascular and all-cause mortality in patients with HF and offers additional information to that provided by NT-proBNP.

## Introduction

It is estimated that about 26 million adults worldwide suffer from heart failure (HF), a number that is expected to increase in the coming decades (1, 2). Despite advances in medical therapy, there has been only a modest improvement in the survival rate of HF in the 21st century, with both 1-year readmission and mortality rates ranging from 30 to 50% and the 5-year mortality reaching 50% (3, 4). Thus, there is an urgent need to establish an individualized therapeutic approach in HF patients, which has been boosted by the availability of biomarker and relative mechanisms-guided management in prognostication, diagnosis, and treatment (5, 6). The most commonly used indicator for HF diagnosis and prognosis is N-terminal pro-B-type natriuretic peptide (NT-proBNP) since it has a longer plasma half-life and less biological variation than those of BNP (7). In addition to NT-proBNP, several other biomarkers possess prognostic value in patients with HF; cystatin C (Cys-C), and prealbumin (PAB) have been extensively studied.

Cys-C, a small 13-kDa endogenous cysteine proteinase inhibitor, is constitutively produced by all nucleated cells. In the kidney, Cys-C is removed from circulation by glomerular filtration and subsequently reabsorbed and catabolized by the proximal convoluted tubules (8). Given this background, it has been proposed that the level of circulating Cys-C may be an appropriate early biomarker of renal impairment. It has also been shown that Cys-C is associated with remodeling of the heart extracellular matrix (9). In addition, it has been revealed that blood and/or myocardium from different animal models of cardiac injury exhibit excessive Cys-C levels (9, 10). Clinically, plasma Cys-C levels are correlated with diastolic dysfunction, left ventricular hypertrophy (LVH), and mortality in HF patients (11, 12). Several studies have reported that baseline Cys-C levels can play a prognostic role in rehospitalization and all-cause mortality in acute decompensated HF (13–15) and chronic heart failure (CHF) (16, 17).

PAB is a visceral protein that exhibits a rapid turnover and reflects the status of whole-body nitrogen metabolism. As a protein with a shorter half-life than that of albumin, PAB can be used as a more precise estimation of a patient's current inflammatory and nutritional status (18), which are associated with a higher mortality rate and longer hospitalization in HF patients (19–21). Additionally, in patients suffering from acute coronary syndrome, lower serum PAB levels (<17 mg/dl) at admission have been shown to be independently predictive of subsequent major adverse cardiac events while hospitalized (22). Moreover, two different studies have demonstrated that low PAB levels (<15 mg/dl) are linked to an increase in short-term mortality and readmission in HF patients (23, 24).

It has been reported that increased Cys-C concentrations are significantly correlated with a higher risk of cardiovascular-related death in the long-term (median follow-up of 2.5 years) (15). A positive correlation has also been found between Cys-C levels and 5-year all-cause mortality in patients with CHF (25), however, an association between low PAB levels and long-term prognosis in HF patients has not yet been revealed. In the present study, we hypothesized that the Cys-C/PAB ratio would be a significant biomarker for the prediction of long-term outcome in HF patients, and combining this ratio with NT-proBNP would further improve risk stratification.

## Materials and Methods

### Study Cohort

Retrospective clinical data were collected from HF patients hospitalized in the Department of Cardiology, Shengjing Hospital of China Medical University, Shenyang, China, between January 2013 and December 2018, with which we established a database. HF was diagnosed according to signs and symptoms, echocardiography, and the results of laboratory tests, as recommended by current guidelines. Heart failure with reduced ejection fraction (HFrEF) was defined as left ventricular ejection fraction (LVEF) <40%, HF with mid-range LVEF (HFmrEF) as 40% ≤ LVEF <50%, and HF with preserved LVEF (HFpEF) as LVEF ≥ 50% (26). In accordance with the cardiac function classification published by the New York Heart Association (NYHA), heart function was divided into four levels (I–IV). Patients displaying evidence of acute myocardial infarction, renal failure, severe anemia, or severe infection were excluded. The follow-up was assessed in December 2020. Patients' survival status was investigated using the population death information registration management system of the Disease Control and Prevention Center of Liaoning Province, and cardiac and non-cardiac mortality was determined in accordance with the International Classification of Diseases (ICD) code of death diagnosis. When information was not available in the system, it was obtained from medical records, patients' physicians, or patients' relatives via telephone. Efforts were made to determine the nature of death in each case. Median follow-up time were 3.3 years (2–8 years). This study was approved by the Shengjing Hospital of China Medical University Ethics Committee and carried out in accordance with the Declaration of Helsinki.

### Variables and Biomarker Assay

The investigators extracted comprehensive clinical data from electronic medical records. The obtained variables included patient demographics, past cardiac and non-cardiac history, physical examination results, laboratory test results, and echocardiography. The laboratory tests of fasting peripheral venous blood samples taken on the day of admission or on the next morning included the following: white blood cell (WBC) counts, platelet counts; and levels of albumin, prealbumin, hemoglobin, glycated hemoglobin, triglyceride, low-density lipoprotein (LDL-C), blood urea nitrogen (BUN), creatinine, uric acid, potassium ion (K^+^), serum sodium ion (Na^+^), troponin I (cTNI), and NT-proBNP. Venous blood samples were tested within 2 h of blood collection in all cases. A particle-enhanced immunonephelometric assay was employed using a Beckman AU 5800 analyzer (Beckman Coulter, Brea, CA, USA) to measure Cys-C and PAB levels. The reference ranges for serum Cys-C and PAB concentrations were 0.59-1.03 mg/L and 18-45 mg/dl, respectively. Left ventricular ejection fraction (LVEF) was determined by echocardiography using the biplane Simpson method within 3 days of admission (27).

### Statistical Analysis

Quantitative variables that normally distributed were compared using one-way analysis of variance and are expressed as the mean ± standard deviation (SD). Quantitative variables with a non-normal distribution were compared using the Mann–Whitney *U*-test and are expressed as the median (interquartile range, IQR). Categorical variables were compared using the chi-square test and are presented as counts and proportions (%). The association of variables with survival was assessed using univariate and multivariable Cox regression models and reported as the hazard ratio (HR) [95% confidence interval (CI)]. We used C statistics to quantify the ability of Cys-C/PAB ratio to identify patients who died, in addition, continuous net reclassification improvement (NRI), and integrated discrimination improvement (IDI) were performed to assess the incremental prognostic value of Cys-C/PAB ratio (28). Patients were allocated to three groups according to tertiles of the Cys-C/PAB ratio and NT-proBNP level (low, medium, high). These two indexes were combined to compare and analyze the HR values. We assigned 0, 1, or 2 points to members of the tertiles of the Cys-C/PAB ratio and NT-proBNP level, with a maximum score of 4. Effects of the Cys-C/PAB ratio and NT-proBNP levels on survival were visualized using Kaplan-Meier curves, and the log-rank test was used to make comparisons. According to the AUROC curve, the Cys-C/PAB ratio in combination with the NT-proBNP level was predictive of cardiovascular and all-cause mortality in HF patients with different types of LVEF. All tests were two-sided, with *P* < 0.05 indicating statistical significance. The Medcalc, and SPSS version 23.0 and SAS9.4 software were used for statistical analysis.

## Results

### Baseline Characteristics

Our cohort retrospectively included 7,563 HF patients hospitalized from January 2013 to December 2018. Those with acute myocardial infarction, severe anemia, renal failure, and severe infection were excluded from this study (Supplementary Figure 1), resulting in a final cohort of 6,311 patients consisting of 2,104 with a low ( ≤ 0.061) Cys-C/PAB ratio, 2,104 with a medium (>0.061 but ≤ 0.102) Cys-C/PAB ratio, and 2103 with a high (>0.102) Cys-C/PAB ratio. Baseline patient characteristics and the occurrence of risk factors according to tertiles of the Cys-C/PAB ratio are shown in Table 1.

**Table 1:** Baseline clinical characteristics, median (IQR), or *N* (%), or means ± SD.

**Variable**	**Cys-C/PAB 1st tertile (≤0.0061)**	**Cys-C/PAB 2st tertile (0.0061-0.0102)**	**Cys-C/PAB 3st tertile (>0.1420)**	***P*-value**
***N***	2,104	2,104	2,103	
**Age (years)**	63.9 ± 13.60	70.2 ± 14.40	73.0 ± 12.63	<0.001
**Male sex**, ***n*** **(%)**	1,207 (57.4)	1,107 (52.6)	1075 (51.1)	<0.001
**NYHA**				<0.001
**NYHA class II**, ***n*** **(%)**	743 (35.3)	347 (16.5)	164 (7.8)	
**NYHA class III**, ***n*** **(%)**	841 (40.0)	922 (43.8)	822 (39.1)	
**NYHA class IV**, ***n*** **(%)**	520 (24.7)	835 (39.7)	1,117 (53.1)	
**CAD**, ***n*** **(%)**	1,337 (63.5)	1,383 (65.7)	1,374 (65.3)	0.286
**Hypertension**, ***n*** **(%)**	1,282 (60.9)	1,324 (62.9)	1,325 (63.0)	0.290
**Diabetes mellitus**, ***n*** **(%)**	610 (29.0)	671 (31.9)	797 (37.9)	<0.001
**Atrial fibrillation**, ***n*** **(%)**	780 (37.1)	645 (30.7)	574 (27.3)	<0.001
**Previous MI**, ***n*** **(%)**	353 (16.8)	423 (20.1)	483 (23.0)	<0.001
**COPD**, ***n*** **(%)**	324 (15.4)	356 (16.9)	361 (17.2)	0.247
**Stroke**, ***n*** **(%)**	356 (16.9)	393 (18.7)	449 (21.4)	0.001
**SBP, mmHg**	136 ± 21.5	135 ± 23.8	135 ± 26.1	0.952
**DBP, mmHg**	82 ± 14.0	81 ± 14.5	79 ± 14.3	<0.001
**Heart rate, b.p.m**.	87 ± 22.5	89 ± 23.1	89 ± 23.1	0.004
**WBC (10**^**∧**^**12/L)**	7.3 ± 2.28	7.3 ± 2.40	7.7 ± 3.10	<0.001
**Hemoglobin (g/L)**	137 ± 17.6	129 ± 19.7	116 ± 23.9	<0.001
**Platelet (10**^**∧**^**9/L)**	198 ± 53.1	190 ± 56.9	185 ± 70.1	<0.001
**Albumin (g/L)**	39.5 ± 3.58	37.2 ± 3.70	34.2 ± 4.55	<0.001
**Prealbumin (mg/dl)**	24.1 ± 5.17	18.8 ± 4.47	14.2 ± 5.86	<0.001
**LDL-C (mmol/L)**	2.8 ± 0.91	2.6 ± 0.92	2.3 ± 0.98	<0.001
**triglycerides (mmol/L)**	1.5 ± 1.15	1.2 ± 0.88	1.1 ± 0.91	<0.001
**HbA1c%**	6.4 ± 1.25	6.5 ± 1.18	6.6 ± 1.26	0.002
**Cys-C (mg/L)**	1.1 ± 0.24	1.5 ± 0.39	2.3 ± 1.02	<0.001
**BUN (mmol/L)**	6.6 ± 2.28	8.2 ± 3.49	12.0 ± 6.96	<0.001
**Creatinine (μmol/L)**	75.6 ± 21.52	91.6 ± 34.66	135.3 ± 80.68	<0.001
**Uric acid (μmol/L)**	394.7 ± 126.05	445.0 ± 147.44	505.2 ± 168.18	<0.001
**Potassium (mmol/L)**	4.0 ± 0.42	4.0 ± 0.50	4.2 ± 0.68	<0.001
**Sodium (mmol/L)**	139.8 ± 3.34	139.0 ± 3.67	137.6 ± 4.63	<0.001
**Troponin I (ug/L)**	0.02 (0.00,0.05)	0.03 (0.01,0.09)	0.05 (0.02,0.17)	<0.001
**NT-proBNP (ng/L)**	1,622 (646,3,499)	3,502 (1,537,7,190)	6,817 (3,295,13,668)	<0.001
**LVEDV (ml)**	159 ± 63.4	160 ± 61.4	170 ± 67.0	0.001
**LVESV (ml)**	84 ± 52.0	89 ± 49.0	93 ± 55.2	<0.001
**LVEF (%)**	50 ± 12.3	47 ± 12.3	47 ± 12.3	<0.001

*NYHA class, New York Heart Association (NYHA) class; CAD, Coronary artery disease; Previous MI, Previous myocardial infarction; COPD, Chronic obstructive pulmonary disease; SBP, Systolic blood pressure; DBP, Diastolic blood pressure; WBC, White blood cells; LDL-C, Low density lipoprotein cholesterol; Cys-C, Cystatin C; NT-proBNP, N-terminal pro–B-type natriuretic peptide; LVEDV, Left ventricular end-diastolic volume; LVESV, Left ventricular end-systolic volume; LVEF, Left ventricular ejection fraction*.

### Clinical Outcome

The primary endpoint of all-cause mortality was reached in 2,945 (46.7%) HF patients during the follow-up, which included 2,071 (32.8%) cardiovascular-related deaths. Univariate Cox regression showed that the Cys-C/PAB ratio was significantly correlated with survival [HR: 1.34 (95% CI, 1.32–1.37), all-cause mortality; HR: 1.33 (95% CI, 1.30–1.37), cardiovascular mortality]. In the multivariable model, following adjustment for age, sex, NYHA class, CAD, Hypertension, Diabetes mellitus, Atrial fibrillation, Previous MI, COPD, SBP, DBP, Heart rate, WBC, Hemoglobin, Platelet, Albumin, LDL-C, Triglycerides, HbA1c,BUN, Creatinine, Uric acid, Potassium, Sodium, Troponin I, LVEDV, LVESV, LVEF. The Cys-C/PAB ratio remained a significant predictive factor of all-cause [adjusted HR: 1.19 (95% CI, 1.16–1.23) and cardiovascular (adjusted HR: 1.19 (95% CI, 1.13–1.24)] mortality (Table 2). Our findings also revealed significant associations between NT-proBNP levels and cardiovascular and all-cause mortality (Table 2).

**Table 2:** The univariate and multivariable Cox regression.

**Variable**	***Univariate analysis***	***P***	Multivariable analysis^*^	***P***
**Cys-C/PAB ratio (per 0.1 increase)**				
Cardiovascular mortality	1.33 (1.30–1.37)	<0.001	1.19 (1.13–1.24)	<0.001
All-cause mortality	1.34 (1.32–1.37)	<0.001	1.19 (1.16–1.23)	<0.001
**Cys-C (per 1 increase)**				
Cardiovascular mortality	1.60 (1.54–1.67)	<0.001	1.27 (1.17–1.38)	<0.001
All-cause mortality	1.61 (1.56–1.66)	<0.001	1.27 (1.18–1.36)	<0.001
**PAB (per 1 increase)**				
Cardiovascular mortality	0.95 (0.94–0.96)	<0.001	0.97 (0.96–0.98)	<0.001
All-cause mortality	0.95 (0.94–0.95)	<0.001	0.97 (0.96–0.98)	<0.001
**NT-proBNP (per 1,000 increase)**				
Cardiovascular mortality	1.08 (1.07–1.08)	<0.001	1.03 (1.02–1.04)	<0.001
All-cause mortality	1.07 (1.06–1.07)	<0.001	1.03 (1.02–1.03)	<0.001

* *adjusted for age, sex, NYHA class, CAD, Hypertension, Diabetes mellitus, Atrial fibrillation, Previous MI, COPD, SBP, DBP, Heart rate, WBC, Hemoglobin, Platelet, Albumin, LDL-C, Triglycerides, HbA1c,BUN, Creatinine, Uric acid, Potassium, Sodium, Troponin I, LVEDV, LVESV, LVEF*.

### Additive Prognostic Value of the Cys-C/PAB Ratio to NT-proBNP Level

**Figure 1:**
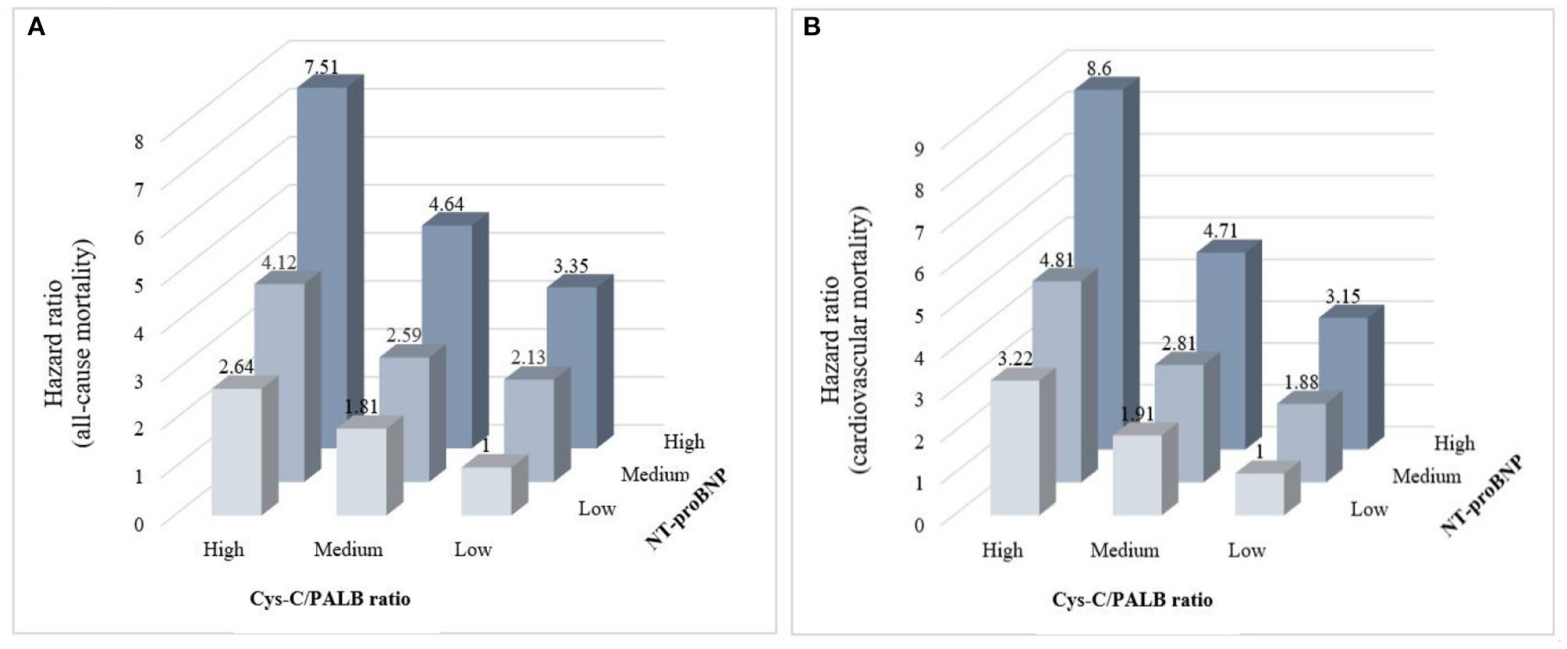
Relative risk stratified by combined tertiles of Cys-C/PAB ratio and NT-proBNP for all-cause mortality **(A)** and cardiovascular mortality **(B)**.

**Figure 2:**
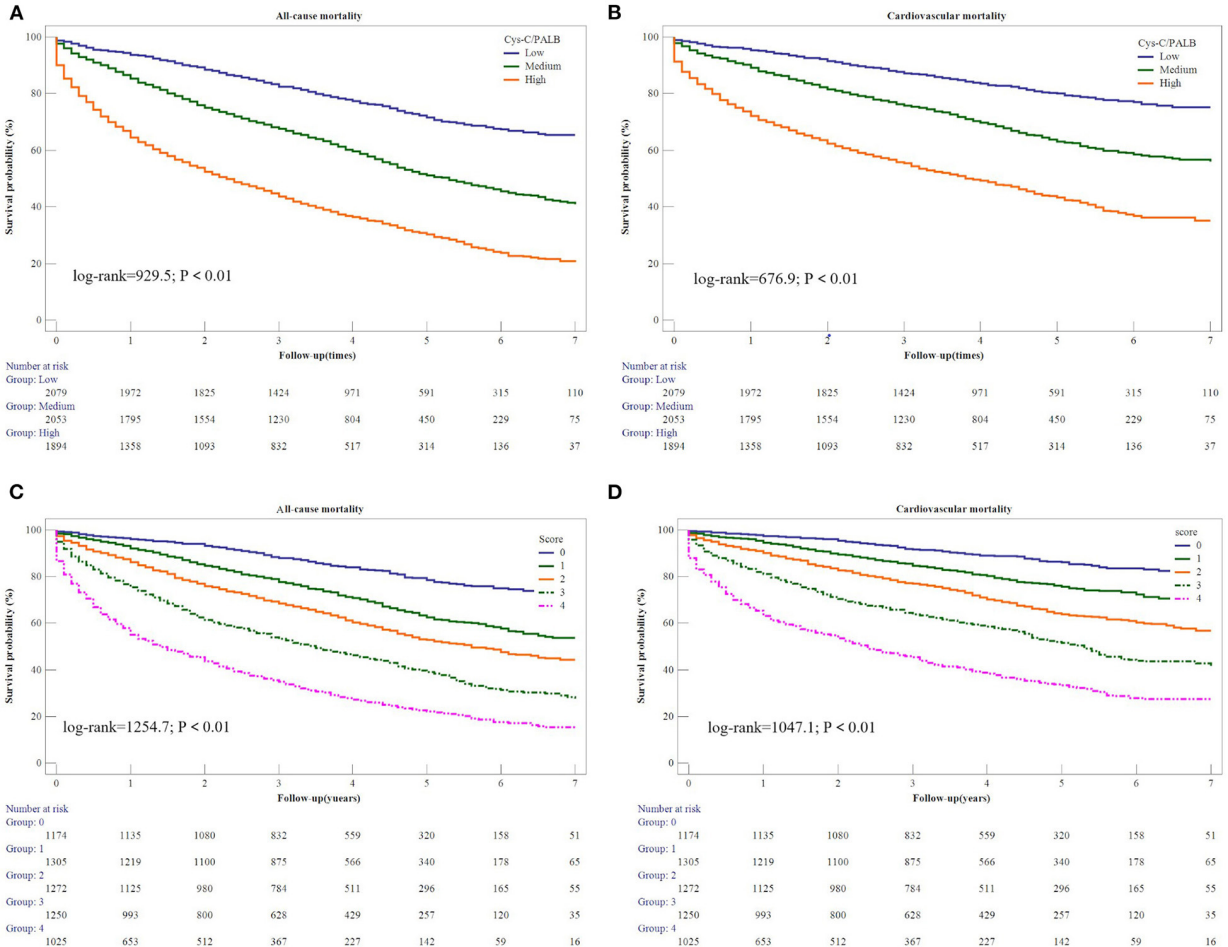
Kaplan-Meier estimates of all-cause mortality **(A)** and cardiovascular mortality **(B)** based on Cys-C/PAB ratio. Kaplan-Meier estimates of all-cause mortality **(C)** and cardiovascular mortality **(D)** based on scores of Cys-C/PAB ratio and NT-proBNP.

The AUROC and integrated discrimination improvement revealed that the Cys-C/PAB ratio significantly improved the prediction of the individual risk of cardiovascular and all-cause mortality when added to NT-proBNP levels (Table 3). Patients were stratified into nine groups according to their tertiles of the Cys-C/PAB ratio and NT-proBNP levels with a view to analyzing the potential additive prognostic value of the former. We observed graduated increases in risk for those in the higher tertiles with respect to both markers. The risk ratio for long-term all-cause mortality for patients with a high Cys-C/PAB ratio in combination with high NT-proBNP levels was 7.51-times higher than for those with low levels, and 8.6-times higher for cardiovascular mortality (Figure 1). We assigned 0, 1, or 2 points to members of the tertiles of the Cys-C/PAB ratio and NT-proBNP level, with a maximum score of 4. Figure 2 shows the Kaplan–Meier survival curves and cardiovascular and all-cause mortality according to Cys-C/PAB ratio and scores of Cys-C/PAB ratio combined with NT-proBNP.

**Table 3:** The C-statistic, discrimination, and reclassification.

	**C-statistic**			**NRI**		**IDI**	
	**Z**	**C-statistic**	**P**	**NRI**	**P**	**IDI**	**P**
**All-cause mortality**
*Cys-C/PAB+NT-proBNP vs Cys-C/PAB*	6.551	0.743 vs. 0.720	<0.001	0.156	0.119	0.064	<0.001
*Cys-C/PAB+NT-proBNP vs NT-proBNP*	9.435	0.743 vs. 0.703	<0.001	0.226	0.072	0.028	0.028
**Cardiovascular mortality**
*Cys-C/PAB+NT-proBNP vs Cys-C/PAB*	7.207	**0.715 vs. 0.679**	<0.001	0.206	0.056	0.079	0.024
*Cys-C/PAB+NT-proBNP vs NT-proBNP*	5.075	**0.715 vs. 0.699**	<0.001	0.216	0.078	0.023	0.030

*Bold values indicates comparation of C-statistic values of Cys-C/PAB+NT-proBNP and C-statistic values of NT-proBNP*.

### Prediction of Clinical Outcomes Using the Cys-C/PAB Ratio in Combination With NT-proBNP Levels

The AUC values for the Cys-C/PAB ratio in combination with NT-proBNP levels for predicting in-hospital, 1-, 3-, 5-, and 8-year all-cause mortality were 0.785, 0.768, 0.742, 0.740, and 0.743, respectively (Table 4), while those for predicting cardiovascular mortality were 0.785, 0.766, 0.727, 0.718, and 0.715, respectively. In the subgroup analysis, we found that the Cys-C/PAB ratio in combination with NT-proBNP levels probably had a greater ability to discriminate the risk of mortality for patients with HFpEF than for patients with HFrEF (Table 4).

**Table 4:** The AUC of Cys-C/PAB ratio combined with NT-proBNP for clinical outcomes prediction.

	***HF (6311)***	***HFrEF (1699)***	***HFmrEF (1676)***	***HFpEF (2936)***
In-hospital mortality	220 (3.5%)	63 (3.7%)	86 (5.1%)	71 (2.4%)
Cardiovascular mortality	0.785 (0.755–0.816)	0.777 (0.711–0.843)	0.763 (0.711–0.814)	0.808 (0.758–0.857)
All-cause mortality	0.785 (0.756–0.814)	0.783 (0.721–0.845)	0.760 (0.710–0.809)	0.810 (0.765–0.854)
1-year mortality	1149 (18.2%)	395 (23.2%)	350 (20.9%)	404 (13.8%)
Cardiovascular mortality	0.766 (0.749–0.782)	0.735 (0.705–0.764)	0.737 (0.705–0.769)	0.780 (0.753–0.807)
All-cause mortality	0.768 (0.753–0.783)	0.737 (0.709–0.765)	0.743 (0.714–0.772)	0.789 (0.766–0.812)
3-year mortality	2197 (34.8%)	726 (42.7%)	652 (38.9%)	819 (27.9%)
Cardiovascular mortality	0.727 (0.713–0.740)	0.686 (0.659–0.712)	0.695 (0.667–0.723)	0.742 (0.720–0.764)
All-cause mortality	0.742 (0.729–0.755)	0.703 (0.678–0.728)	0.716 (0.691–0.741)	0.762 (0.743–0.781)
5-year mortality	2767 (43.8%)	895 (52.7%)	799 (47.7%)	1073 (36.5%)
Cardiovascular mortality	0.718 (0.704–0.731)	0.675 (0.649–0.700)	0.692 (0.665–0.719)	0.727 (0.706–0.748)
All-cause mortality	0.740 (0.728–0.752)	0.703 (0.678–0.727)	0.721 (0.697–0.745)	0.753 (0.735–0.771)
8-year mortality	2945 (46.7%)	951 (56.0%)	838 (50%)	1156 (39.4%)
Cardiovascular mortality	0.715 (0.702–0.728)	0.669 (0.643–0.694)	0.691 (0.664–0.717)	0.725 (0.704–0.745)
All-cause mortality	0.743 (0.731–0.755)	0.702 (0.677–0.726)	0.726 (0.702–0.750)	0.756 (0.739–0.774)

## Discussion

Among the 6,311 patients hospitalized due to HF, 2,945 (46.7%) died during the follow-up period (Median 3.3 years, range 2–8 years). Our findings show that cardiovascular and all-cause mortality in patients with HF could be predicted independently by both high Cys-C levels and low PAB levels. Thus, it is unsurprising that a higher Cys-C/PAB ratio provided great value for predicting all-cause and cardiovascular mortality. When this ratio was combined with NT-proBNP, even better prognostic prediction was achieved in HF patients in the long term. Our results show that the risk ratio of long-term all-cause mortality for patients with a high Cys-C/PAB ratio in combination with high NT-proBNP levels was 7.51-times higher than for those with low levels, and 8.6-times for cardiovascular mortality. This study also shows high value of the Cys-C/PAB ratio in combination with NT-proBNP levels for predicting in-hospital or long-term all-cause and cardiovascular mortality in HF patient.

HF is a complex syndrome involving different pathophysiological pathways (e.g., remodeling, myocardial injury, fibrosis, and inflammation), the components of which can be reflected by various biomarkers, including natriuretic peptides, PAB, galectin-3, soluble suppressor of tumorgenicity 2 (ST-2), Cys-C, interleukin-6 (IL-6), highly sensitive troponin, and procalcitonin (21, 29). These pathways may provide biomarkers that could act as a clinical bridge between HF and potential treatment strategies (5). *In vitro*, cardiomyocytes and fibroblasts release an excess of Cys-C upon exposure to oxidative stress, which has also been confirmed by *in vivo* studies (9), and Cys-C can in turn promote cardiomyocyte injury and autophagy (30, 31) under oxidative stress. In addition, an increase in Cys-C may be positively associated with osteopontin, a profibrotic matricellular protein associated with myocardial fibrosis in HF patients (32). It may also inhibit the degradation of tissue inhibitor of metalloproteinases-1 (TIMP-1) and osteopontin, promoting myocardial fibrosis and aggravating ventricular remodeling, which leads to a vicious cycle (33). Studies have also demonstrated that Cys-C can selectively inhibit the activity of cystine protease, reduce elastic fiber degradation in cardiomyocytes, and increase the destruction of myocardial collagen fibers, disturbing cardiac structure and function (34, 35). These mechanisms may explain why clinical studies have shown a significant association between Cys-C and diastolic dysfunction and left ventricular hypertrophy (LVH) in HF patients, in addition to other indicators of renal function such as eGFR and serum creatinine (12, 36).

Malnutrition is commonly found in patients suffering from chronic conditions, including HF. Its prevalence in HF patients has been reported to vary from 20 to 70% (37). Albumin, a well-established prognostic factor in patients with HF, is a biomarker reflecting nutritional status, while with a half-life up to 17 days (38), it is insensitive to changes of nutritional status. PAB, known as a transthyroxine protein, is a complex molecule of a non-glycosylated protein and a retinol-binding protein. The reduced sensitivity to hydration status, small pool, and short half-life (2 days) promotes its use in detecting early deficits in nutritional status (39). Thus, PAB is now accepted as a more accurate biomarker of nutritional status, with a higher sensitivity than albumin to changes in nutrition. PAB is an acute-phase protein whose levels are decreased under inflammatory conditions due to cytokine stimulation (40). Therefore, in addition to being a nutritional marker, PAB may be an inflammatory reactant during the acute stage (23, 41). Franco et al. showed a significant correlation of high CRP levels with low PAB levels in patients with acute HF, indicating that systemic inflammation may also occur in patients suffering from protein malnutrition (23), contributing to a worse outcome.

### Clinical Implications

Regarding the future application of prognostic biomarkers, guidance for therapy and rehabilitation is potentially the most valuable use. It should be borne in mind that changes in biomarkers themselves are not as important as determinants of the outcome; instead, their cause and the clinical context during which these changes develop are most important. As suggested by D'Elia et al. (42), biomarkers may act as a clinical bridge between the pathophysiological changes in HF and potential treatment strategies. As mentioned above, there are associations between Cys-C and ventricular remodeling and myocardial fibrosis, and therapeutic strategies aimed at reducing these processes may achieve certain cardioprotective results in patients with HF. In fact, Lopez et al. showed that treatment with various diuretics that act at the ascending limb of the loop of Henle may have different long-term effects on myocardial fibrosis in patients suffering from chronic failure. Moreover, patients treated with torasemide, but not those treated with furosemide, displayed decreased type I collagen synthesis and decreased accumulation of myocardial collagen (43). This indicates the need for more sophisticated mechanisms concerning biomarkers and HF pathophysiological changes in order to perform individualized therapy and improve prognosis in HF patients. Since low PAB levels are representative of malnutrition or inflammation, we should clinically assess the intake or absorption of nutrients by taking patient history or providing adequate feeding. If malnutrition is ruled out, treatment of the disease causing the inflammation and excessive cytokine production should be considered (44).

### Limitations

There are a few limitations of this study. Firstly, the single-center retrospective nature of this work confers an inherent limitation associated with retrospective investigation, preventing us from ruling out the effects of residual or unmeasured confounding factors. Retrospective studies are also inherently associated with a risk of selection bias. Secondly, the Cys-C, NT-proBNP and PAB levels were measured at baseline in our study without dynamic monitoring, limiting accuracy. Thirdly, HF is a highly complex syndrome orchestrated by many different pathophysiological pathways. As such, Cys-C, PAB, and NT-proBNP may not provide sufficient prognostic information; thus, further biomarkers reflecting different pathophysiological processes may be required to provide greater prognostic value than their isolated use.

## Conclusions

Our findings reinforce the assumption that the Cys-C/PAB ratio is a valuable predictive factor for cardiovascular and all-cause mortality in patients with HF. Moreover, this ratio provided additional prognostic information alongside NT-proBNP for HF patients.

## Data Availability Statement

The original contributions presented in the study are included in the article/Supplementary Material, further inquiries can be directed to the corresponding author/s.

## Ethics Statement

The study was approved by the Shengjing Hospital of China Medical University Ethics Committee and carried out in accordance with the Declaration of Helsinki. Written informed consent for participation was not required for this study in accordance with the national legislation and the institutional requirements.

## Author Contributions

CW and ZS conceived and designed the study and wrote the paper. CW, SH, and YL extracted and sorted clinical data. ZL and FT analyzed the data. All authors contributed to the article and approved the submitted version.

## Conflict of Interest

The authors declare that the research was conducted in the absence of any commercial or financial relationships that could be construed as a potential conflict of interest.
