# Supplementary figures and images for: Predictive Value of the Serum Cystatin C/Prealbumin Ratio in Combination With NT-proBNP Levels for Long-Term Prognosis in Chronic Heart Failure Patients: A Retrospective Cohort Study
Source: Front Cardiovasc Med. 2021 Jul 14;8:684919. doi: 10.3389/fcvm.2021.684919 (PMC8316687; doi:10.3389/fcvm.2021.684919)

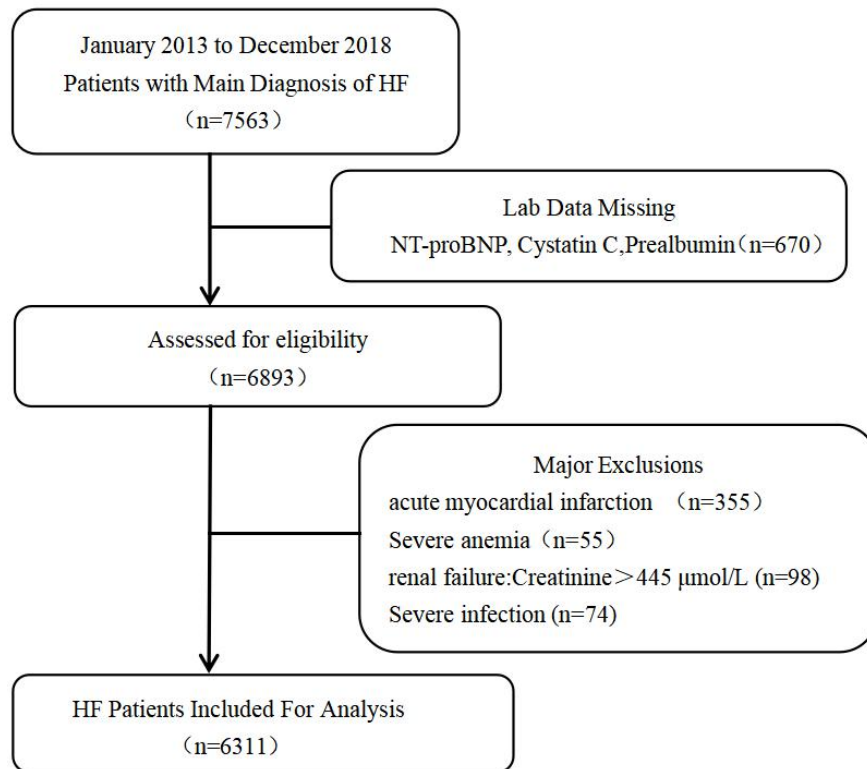

Supplementary Figure 1. Flow diagram of participant selection.

Supplement: Supplementary file 1 [file Data_Sheet_1.PDF]
